# Television Viewing Time is Associated with Overweight/Obesity Among Older Adults, Independent of Meeting Physical Activity and Health Guidelines

**DOI:** 10.2188/jea.JE20110054

**Published:** 2012-01-05

**Authors:** Shigeru Inoue, Takemi Sugiyama, Tomoko Takamiya, Koichiro Oka, Neville Owen, Teruichi Shimomitsu

**Affiliations:** 1 Department of Preventive Medicine and Public Health, Tokyo Medical University, Tokyo, Japan; 2 Baker IDI Heart and Diabetes Institute, Melbourne, Australia; 3 Faculty of Sport Sciences, Waseda University, Tokorozawa, Japan; 4 Cancer Prevention Research Centre, School of Population Health, The University of Queensland, Brisbane, Australia

**Keywords:** sedentary behavior, cardiovascular risk factor, obesity

## Abstract

**Background:**

Previous studies have shown associations of sedentary behavior with cardiovascular risk, independent of moderate-to-vigorous physical activity (MVPA). However, few studies have focused on older adults. This study examined the joint associations of television (TV) viewing time and MVPA with overweight/obesity among Japanese older adults.

**Methods:**

A population-based, cross-sectional mail survey was used to collect self-reported height, weight, time spent in TV viewing, and MVPA from 1806 older adults (age: 65–74 years, men: 51.1%). Participants were classified into 4 categories according to TV viewing time (dichotomized into high and low around the median) and MVPA level (dichotomized into sufficient and insufficient by the physical activity guideline level of ≥150 minutes/week). Odds ratios (ORs) for overweight/obesity (body mass index ≥25 kg/m^2^) were calculated according to the 4 TV/MVPA categories, adjusting for potential confounders.

**Results:**

Of all participants, 20.1% were overweight/obese. The median TV viewing time (25th, 75th percentile) was 840 (420, 1400) minutes/week. As compared with the reference category (high TV/insufficient MVPA), the adjusted ORs (95% CI) of overweight/obesity were 0.93 (0.65, 1.34) for high TV/sufficient MVPA, 0.58 (0.37, 0.90) for low TV/insufficient MVPA, and 0.67 (0.47, 0.97) for low TV/sufficient MVPA.

**Conclusions:**

In this sample of older adults, spending less time watching TV, a predominant sedentary behavior, was associated with lower risk of being overweight or obese, independent of meeting physical activity guidelines. Further studies using prospective and/or intervention designs are warranted to confirm the presently observed effects of sedentary behavior, independent of physical activity, on the health of older adults.

## INTRODUCTION

Sedentary behavior (too much sitting, as distinct from too little exercise) is related to adverse cardiometabolic risk profiles and premature mortality.^1^^–^^10^ Several studies have examined television (TV) viewing time as a predominant sedentary behavior and have shown associations with obesity and other cardiovascular risk factors.^1^^–^^3^^,^^7^^,^^8^^,^^10^ Furthermore, these associations between sedentary behaviors and cardiovascular risk factors were observed regardless at all levels of moderate-to-vigorous physical activity (MVPA), defined by an intensity of 3 metabolic equivalents (METs) or greater. Sugiyama et al^4^ reported that adults who met current physical activity guidelines (MVPA of ≥30 min/day for 5 days/week)^11^^,^^12^ but had high levels of sedentary time were about 1.5 times more likely to be overweight or obese, relative to those who met physical activity guidelines and had lower levels of sedentary time. These findings suggest that prolonged sedentary behavior elevates health risk, independent of MVPA participation.

However, few studies of the associations of sedentary behavior and physical activity with health risk have focused on older adults.^10^ Older adults tend to have lower levels of physical activity^13^^,^^14^ and to spend more time in sedentary behavior.^15^ They also begin to lose fitness levels, and some of them find it difficult to adopt and maintain MVPA.^16^ Because of these changes in the behavior patterns of older adults, it is important to assess how different combinations of sedentary behavior and physical activity might influence their cardiovascular health. It is important to examine if older adults could improve their health by reducing sedentary behavior, that is, by avoiding prolonged sedentary behavior and increasing light-intensity physical activity, regardless of their level of MVPA. Thus, the purpose of this study was to examine the joint associations of TV viewing time and MVPA with overweight/obesity among older adults in Japan.

## METHODS

### Participants and data collection

This cross-sectional study was part of a project to investigate the association between neighborhood environment and physical activity among older adults.^17^ Data were collected from February to March 2010. A total of 2700 residents who were aged 65 to 74 years and living in 3 Japanese cities (Bunkyo Ward in Tokyo, Fuchu in Tokyo, Oyama in Shizuoka prefecture) were randomly selected from registries of residential addresses and stratified by sex, age (65–69 years/70–74 years), and city of residence. In total, 2700 older adults were identified, and they received invitation letters that described the content of the study. Three cities were chosen—one each from a metropolitan urban area, a suburban area, and a rural area—because this survey was originally designed to investigate the relationship of neighborhood environment with physical activity. Bunkyo is in central Tokyo. Fuchu is a suburban city located about 20 km east of the center of Tokyo. It is in the Tokyo Metropolitan Area and within commuting distance of central Tokyo. Oyama is a small regional city located about 80 km west of Tokyo.

Two weeks after the invitation, the 2700 older adults received a questionnaire and consent form. To encourage participation, a 500-yen (about 6 US dollars in 2011) book voucher was offered to respondents. During the survey, a call center was set up to answer participants’ inquiries. Reminders to return the survey were mailed twice to nonresponding participants. Those who returned an incomplete survey were asked to complete the survey again. Of 2700 older adults initially identified, 2046 returned the survey. After data cleaning, 1806 participants had valid data for the analyses of this study (response rate: 66.9%). This study received prior approval from the Tokyo Medical University Ethics Committee.

### Measures

#### Outcome variable

Body mass index (BMI) was the outcome measure of this study and was calculated from self-reported weight and height. Participants were categorized as normal weight (BMI <25 kg/m^2^) and overweight or obese (BMI ≥25 kg/m^2^) for regression analyses.

#### TV viewing time and physical activity

TV viewing time was determined by asking participants to report frequency of TV viewing (days/week) and average viewing time in each day (minutes/day) over the past 7 days. This questionnaire item was a Japanese translation of an Australian questionnaire on leisure-time sedentary behaviors.^18^ TV viewing time was dichotomized using median to high TV viewing (>840 min/week) and low TV viewing (≤840 min/week).

The Short Version of the International Physical Activity Questionnaire (IPAQ-S)^19^^,^^20^ was used to assess moderate-to-vigorous physical activity (MVPA). Among Japanese adults, the test-retest reliability of IPAQ-S (intraclass correlation coefficient: ICC) was 0.87 and its validity as compared with accelerometry (Spearman’s correlation coefficient: ρ) was 0.39.^20^ Among elderly adults, reliability and validity as compared with pedometry (partial correlation coefficient adjusted for sex, age, and education: *r*) were reported only in a Chinese study (ICC = 0.84; *r* = 0.33).^21^ Participants were asked to report the frequency and duration of 3 types of physical activity: vigorous intensity, moderate intensity (excluding walking), and walking. Total time spent in MVPA, including walking, was calculated as the sum of these 3 activities, which was then classified as insufficient MVPA (<150 min/week) and sufficient MVPA (≥150 min/week), using physical activity guidelines for health benefits.^12^^,^^22^

#### Sociodemographic, lifestyle, and health variables

In addition to age and sex, which were obtained from the registry of residential addresses of each city, educational attainment (years of education), working status (working hours per week), smoking habits (currently smoking or not), alcohol consumption (days/week), and physical functioning were assessed by questionnaire. Questions on smoking and alcohol were from the National Health and Nutrition Survey of Japan.^23^ Physical functioning was assessed by using an item in the 8-Item Short-Form Health Survey (SF8).^24^ Participants chose the most suitable response from a 5-point scale to the statement, “During the past 4 weeks, how much did physical health problems limit your usual physical activities (such as walking or climbing stairs)?”. The choices were “not at all”, “very little”, “somewhat”, “quite a lot”, and “could not do physical activity”.

### Statistical analyses

Participants were classified into 4 groups by combinations of TV viewing time and MVPA: high TV/insufficient MVPA, high TV/sufficient MVPA, low TV/insufficient MVPA, and low TV/sufficient MVPA. Logistic regression analyses were used to calculate the odds ratios (ORs) and 95% CIs for being overweight/obese (BMI ≥25 kg/m^2^) by the 4 categories (the reference category was high TV/insufficient MVPA). Two models were examined. Model 1 adjusted for sex and age. Model 2 adjusted for sex, age, education (>12 years; ≤12 years), working status (working for ≥35 hours/week; working for 1–34 hours/week; not working), city of residence (Bunkyo; Fuchu; Oyama), smoking (current smoker; not current smoker), drinking (≥1 day/week; <1 day/week), and physical functioning (5-point scale mentioned above). Analyses were conducted first for the overall sample, then separately for men and women, and for persons who were working (>0 hour/week) and not working, because the time available for leisure activities is likely to differ between these groups. In addition, 2 subsample analyses were conducted to examine potential confounding. First, extremely low physical functioning was a potential confounder. Thus, 40 participants who answered “could not do physical activity” to the question on physical functioning^24^ were excluded from analyses (*n* = 1766). Second, 127 underweight participants (BMI <18.5 kg/m^2^), who were included in the normal reference category in the main analyses, were excluded from the second subsample analyses (*n* = 1679). Significance was considered to be *P* < 0.05. Analyses were conducted with SPSS Version 17.0 for Windows (SPSS Inc., Tokyo, Japan).

## RESULTS

Table 1 shows the characteristics of the study sample. The mean age (SD) was 69.2 (2.9). The prevalence of being overweight/obese was 20.1% among the overall sample. The prevalence of each of the combined categories of TV viewing and MVPA was 24.6% for high TV/insufficient MVPA, 22.1% for high TV/sufficient MVPA, 17.6% for low TV/insufficient MVPA, and 18.0% for low TV/sufficient MVPA. The median (25th, 75th percentile) TV viewing time and MVPA were 840 (420, 1400) min/week and 300 (120, 630) min/week, respectively.

**Table 1.** Characteristics of participants by combined categories of TV viewing time and physical activity

	Overall		High TV viewing/ Insufficient PA^b^		High TV viewing/ Sufficient PA^b^		Low TV viewing/ Insufficient PA^b^		Low TV viewing/ Sufficient PA^b^		*P* value^c^
	*N* = 1806		*N* = 256		*N* = 544		*N* = 262		*N* = 744		
				
	*n*	%	*n*	%	*n*	%	*n*	%	*n*	%	
Sex											
Male	925	51.2	125	48.8	275	50.6	127	48.5	398	53.5	0.389
Female	881	48.8	131	51.2	269	49.4	135	51.5	346	46.5	
Age, years											
Mean (SD)	69.6 (2.9)		70.0 (3.0)		69.4 (2.9)		70.1 (3.0)		69.4 (2.9)		<0.001
City of residence											
Bunkyo	571	31.6	50	19.5	174	32.0	76	29.0	271	36.4	<0.001
Fuchu	626	34.7	74	28.9	188	34.6	77	29.4	287	38.6	
Oyama	609	33.7	132	51.6	182	33.5	109	41.6	186	25.0	
Education, years											
<13	1158	64.1	198	77.3	349	64.2	193	73.7	418	56.2	<0.001
13+	648	35.9	58	22.7	195	35.8	69	26.3	326	43.8	
Working status											
Not working	1110	61.5	200	78.1	363	66.7	163	62.2	384	51.6	<0.001
1–34 hours/wk	409	22.6	26	10.2	119	21.9	53	20.2	211	28.4	
35+ hours/wk	287	15.9	30	11.7	62	11.4	46	17.6	149	20.0	
Current smoking											
Yes	273	15.1	55	21.5	88	16.2	34	13.0	96	12.9	0.006
No	1533	84.9	201	78.5	456	83.8	228	87.0	648	87.1	
Drinking, days/week											
1+	725	40.1	88	34.4	216	39.7	87	33.2	334	44.9	0.001
<1	1081	59.9	168	65.6	328	60.3	175	66.8	410	55.1	
Limitation of physical functioning											
Not at all	1086	60.1	122	47.7	348	64.0	119	45.4	497	66.8	<0.001
Very little	337	18.7	49	19.1	99	18.2	55	21.0	134	18.0	
Somewhat	258	14.3	48	18.8	80	14.7	46	17.6	84	11.3	
Quite a lot	85	4.7	22	8.6	14	2.6	25	9.5	24	3.2	
Could not do physical activity	40	2.2	15	5.9	3	0.6	17	6.5	5	0.7	
BMI, kg/m^2^											
<25	1443	79.9	193	75.4	424	77.9	216	82.4	610	82.0	0.055
25+	363	20.1	63	24.6	120	22.1	46	17.6	134	18.0	
TV viewing, min/week											
Short, ≤840	1006	55.7	0	0.0	0	0.0	262	100.0	744	100.0	<0.001
Long, 840+	800	44.3	256	100.0	544	100.0	0	0.0	0	0.0	
Median (25%tile, 75%tile)	840 (420, 1400)		1680 (1260, 2520)		1500 (1260, 2100)		420 (150, 840)		480 (255, 840)		
MVPA^a^, min/week											
Insufficient, <150	518	28.7	256	100.0	0	0.0	262	100.0	0	0.0	<0.001
Sufficient, 150+	1288	71.3	0	0.0	544	100.0	0	0.0	744	100.0	
Median (25%tile, 75%tile)	300 (120, 630)		20 (0, 90)		420 (272.5, 750)		20 (0, 80)		480 (300, 840)		

^a^MVPA: moderate-to-vigorous physical activity.

^b^TV viewing time was dichotomized by the median (840 min/wk); physical activity (PA) was dichotomized by MVPA of 150 min/wk.

^c^Differences between groups were examined by chi-square tests for categorical variables and 1-way analysis of variance for age.

Table 2 shows the ORs for being overweight/obese, according to the 4 TV viewing/MVPA categories. For the overall sample, those who belonged to the most active category (low TV/sufficient MVPA) were significantly less likely to be overweight/obese, in comparison with the reference group (high TV/insufficient MVPA), after adjusting for sex and age (Model 1). After further adjustment for other potential confounders (Model 2), the 2 low-TV categories had significantly lower odds ratios of overweight/obesity: the ORs (95% CI) were 0.58 (0.37, 0.90) for low TV/insufficient MVPA and 0.67 (0.47, 0.97) for low TV/sufficient MVPA. No significant association was observed for the high TV/sufficient MVPA category (OR: 0.93 [0.65, 1.34]).

**Table 2.** Odds ratios for overweight/obesity by the combined categories of TV viewing time and physical activity

TV/PA categories^a^	Sample	Overweight /obesity, %	Model 1^b^		Model 2^c^	
	
			OR (95% CI)	*P* value	OR (95% CI)	*P* value
Overall						
High TV/ ​ Insufficient PA	256	24.6	1.00		1.00	
High TV/ ​ Sufficient PA	544	22.1	0.85 (0.60, 1.21)	0.370	0.93 (0.65, 1.34)	0.693
Low TV/ ​ Insufficient PA	262	17.6	0.65 (0.43, 1.00)	0.052	0.58 (0.37, 0.90)	0.014
Low TV/ ​ Sufficient PA	744	18.0	0.65 (0.46, 0.92)	0.015	0.67 (0.47, 0.97)	0.033
Men						
High TV/ ​ Insufficient PA	125	24.8	1.00		1.00	
High TV/ ​ Sufficient PA	275	26.2	1.05 (0.64, 1.71)	0.845	1.05 (0.63, 1.75)	0.846
Low TV/ ​ Insufficient PA	127	18.1	0.68 (0.37, 1.25)	0.215	0.54 (0.29, 1.02)	0.057
Low TV/ ​ Sufficient PA	398	20.9	0.77 (0.48, 1.24)	0.281	0.69 (0.42, 1.15)	0.154
Women						
High TV/ ​ Insufficient PA	131	24.4	1.00		1.00	
High TV/ ​ Sufficient PA	269	17.8	0.68 (0.41, 1.13)	0.136	0.83 (0.49, 1.40)	0.484
Low TV/ ​ Insufficient PA	135	17.0	0.63 (0.35, 1.16)	0.138	0.59 (0.32, 1.10)	0.099
Low TV/ ​ Sufficient PA	346	14.7	0.54 (0.33, 0.89)	0.015	0.66 (0.39, 1.11)	0.120
Working						
High TV/ ​ Insufficient PA	56	23.2	1.00		1.00	
High TV/ ​ Sufficient PA	181	24.3	1.05 (0.52, 2.14)	0.891	1.17 (0.56, 2.45)	0.681
Low TV/ ​ Insufficient PA	99	20.2	0.87 (0.39, 1.92)	0.722	0.73 (0.32, 1.67)	0.452
Low TV/ ​ Sufficient PA	360	22.8	0.95 (0.49, 1.86)	0.883	1.04 (0.51, 2.12)	0.906
Not working						
High TV/ ​ Insufficient PA	200	25.0				
High TV/ ​ Sufficient PA	363	20.9	0.79 (0.53, 1.20)	0.269	0.89 (0.59, 1.36)	0.601
Low TV/ ​ Insufficient PA	163	16.0	0.57 (0.34, 0.97)	0.038	0.55 (0.33, 0.94)	0.030
Low TV/ ​ Sufficient PA	384	13.5	0.47 (0.31, 0.73)	<0.001	0.54 (0.34, 0.84)	0.007

^a^TV viewing time was dichotomized by the median (840 min/wk); physical activity (PA) was dichotomized by MVPA of 150 min/wk.

^b^Model 1: adjusted for sex and age.

^c^Model 2: adjusted for sex, age, education, employment status, city of residence, smoking, drinking, and physical functioning, excluding stratified variables.

When men and women were examined separately, a significant association between TV/MVPA category and overweight was observed in the low TV/sufficient MVPA among women in Model 1, and borderline significant associations in the low TV/insufficient PA category among men and women were observed in Model 2. In the stratified analyses by working status, a significant association between TV/MVPA category and overweight was observed only among nonworking older adults. The ORs of being overweight, after adjusting for all covariates, were 0.89 (0.59, 1.36) for high TV/sufficient MVPA, 0.55 (0.33, 0.94) for low TV/insufficient MVPA, and 0.54 (0.34, 0.84) for low TV/sufficient MVPA.

The findings from 2 subsample analyses (a sample excluding those with poor physical function and one without underweight participants) showed a similar pattern: a significantly lower odds of being overweight was observed in the low TV/insufficient MVPA and low TV/sufficient MVPA categories (data not shown).

In addition, because the prevalence of meeting physical activity guidelines was high (71.3%), we conducted analyses using a different cut point for MVPA (median: 300 min/wk; data not shown in the tables) to examine the potential influence of overestimation. However, this did not substantially change the overall pattern of findings. Those in the category of “low TV viewing/insufficient PA (≤300 min/wk)” and “low TV viewing/sufficient PA (>300 min/wk)” had lower risk of overweight/obesity (OR: 0.66 [0.48–0.92], 0.67 [0.48–0.93], respectively), while no significant association between the risk of overweight/obesity and the category of “high TV viewing/sufficient PA (>300 min/wk)” was observed.

## DISCUSSION

This study found that older adults who spent less time watching TV, a predominant leisure-time sedentary behavior, were less likely to be overweight or obese, regardless of their levels of MVPA. This suggests that prolonged TV viewing elevates the risk of overweight/obesity among the elderly population. Analyses also suggested that in the presence of prolonged TV viewing, a sufficient amount of MVPA, as defined by current physical activity guidelines, was not protective against overweight/obesity in this study sample. These findings could be interpreted as suggesting the importance of light-intensity activity to reduce obesity risk. A previous study showed that light-intensity activity, which is negatively correlated with sedentary time, had beneficial associations with cardiometabolic biomarkers.^25^ Because some older people have difficulty in adopting and maintaining MVPA,^16^ reducing sedentary behavior and increasing light-intensity activity may be an effective and practical strategy to achieve health benefits in this age group.

Associations of sedentary behavior, including TV viewing time, with obesity measures, independent of MVPA, have been consistently reported for adult samples.^2^^–^^7^^,^^9^ Our study found that this was also the case with older adults. However, our study was slightly different from previous studies in that the association of MVPA with overweight/obesity seemed weaker than that of TV viewing time. A previous study on adults reported that those who spent more time in sedentary behaviors (but were sufficiently physically active) and those who were insufficiently active (but spent less time in sedentary behavior) had similar risks of overweight.^4^ In youth studies, insufficient physical activity was more strongly associated than prolonged sedentary behavior with overweight.^26^^,^^27^ In light of these previous studies, it is possible to argue that the impact of sedentary behavior and MVPA on obesity risk differs with age and that prolonged sedentary behavior might be a stronger risk factor for elderly adults. The association between sedentary behavior and cardiovascular risk will be influenced by non-exercise activity thermogenesis (NEAT), which is generally a much greater component of total energy expenditure than MVPA, and by the significant role of brief yet frequent muscle contractions throughout the day, which may short-circuit unhealthy molecular signals that cause metabolic dysfunction.^28^ These effects might be more pronounced among older adults, who are generally less physically active than younger adults.

The fact that significant associations of TV viewing time with overweight were found in nonworkers but not in workers suggests that light-intensity and intermittent activities during work are protective against overweight/obesity in the presence of prolonged TV viewing time. However, in nonworkers, some TV viewing may accompany other leisure-time sedentary behaviors, due to the greater amount of time available for them. An Australian study found that TV viewing time was a good marker of overall sedentary time.^29^ Our findings suggest that retired older adults are at risk of overweight. Thus, retirement might be a window of opportunity for interventions that prevent and reduce sedentary time.

There are some limitations that need to be considered in interpreting the findings of this study. First, both the dependent and independent variables were measured by self-report, which is susceptible to response bias. In particular, the percentage of participants who met physical activity guidelines was high. Overestimation of physical activity may have contributed to the weaker association of MVPA and overweight observed in this study. Although an additional analysis using a different cut point (median, 300 min/wk) produced a similar pattern of findings, reporting error and bias may have masked associations of MVPA with overweight. In addition, we used BMI calculated from self-reported weight and height. Although self-reported measurement generally has a high correlation with direct measurement,^30^^–^^35^ some studies have suggested that obese and elderly persons tend to underreport their weight.^30^^–^^35^ If participants tend to report their behavior and weight biased to the optimal direction, this may have reduce response variability and lead to lower statistical power and underestimation of associations.^33^ Future studies should use objective measures of behaviors and overweight/obesity, to more accurately assess the health effects of sedentary behaviors among older adults. The cross-sectional design of this study is another limitation, and the possibility of reverse causality (ie, overweight and obesity could discourage activity and lead to prolongation of TV viewing time) should be considered. Longitudinal studies are needed to examine causality. Finally, the analyses could not include information on diet, which may confound the relationship between sedentary time and overweight risk.

In spite of these limitations, the current study adds new findings on the associations between TV viewing time and overweight/obesity independent of MVPA among older adults, especially among those not working. As people get older, they typically become less active and spend more time in sedentary behaviors. Further research examining the relative importance of sedentary behavior and physical activity on health outcomes is thus warranted to inform the development of public health initiatives and guidelines for older people.
